# HTS-Net: An integrated regulome-interactome approach for establishing network regulation models in high-throughput screenings

**DOI:** 10.1371/journal.pone.0185400

**Published:** 2017-09-26

**Authors:** Claire Rioualen, Quentin Da Costa, Bernard Chetrit, Emmanuelle Charafe-Jauffret, Christophe Ginestier, Ghislain Bidaut

**Affiliations:** 1 Aix-Marseille Univ, Marseille, France; 2 Inserm, U1068, Centre de Recherche en Cancérologie de Marseille, Marseille, France; 3 Institut Paoli-Calmettes, Marseille, France; 4 CNRS, UMR7258, Centre de Recherche en Cancérologie de Marseille, Marseille, France; Instituto Nacional de Medicina Genomica, MEXICO

## Abstract

High-throughput RNAi screenings (HTS) allow quantifying the impact of the deletion of each gene in any particular function, from virus-host interactions to cell differentiation. However, there has been less development for functional analysis tools dedicated to RNAi analyses. HTS-Net, a network-based analysis program, was developed to identify gene regulatory modules impacted in high-throughput screenings, by integrating transcription factors-target genes interaction data (regulome) and protein-protein interaction networks (interactome) on top of screening z-scores. HTS-Net produces exhaustive HTML reports for results navigation and exploration. HTS-Net is a new pipeline for RNA interference screening analyses that proves better performance than simple gene rankings by z-scores, by re-prioritizing genes and replacing them in their biological context, as shown by the three studies that we reanalyzed. Formatted input data for the three studied datasets, source code and web site for testing the system are available from the companion web site at http://htsnet.marseille.inserm.fr/. We also compared our program with existing algorithms (CARD and hotnet2).

## Introduction

*In vitro* functional studies using RNA interference (RNAi) screening libraries have recently dramatically improved in throughput speed, quality and genomic coverage with the advent of powerful biochemical methods for perturbing genes transcriptional mechanisms. RNAi screenings and the construction of associated genome-wide small interfering RNA (siRNA) libraries allowed a refined understanding of gene function at the genomic scale. In parallel, the improvement of analytical microscopy and the development of high-content screening tools (HCS) have allowed scientists to access multi-parametric analyses at a single-cell level. Together, these technologies charted the way to high-throughput screenings (HTS) with sophisticated cellular read-outs at the genomic scale. The hits detected by such assays can quickly link single proteins to a studied phenotype/function [1][2].

Primary data analysis such as normalization [3], quality assessment, and hits selection [4] is well addressed in the literature, and stable software have started to emerge. While classic statistics are widely used for reporting hits values (z-scores, signal-to-noise ratios), new statistics were proposed, such as the strictly standardized mean difference (SSMD) measurement [5]. Goktug and colleagues [6] proposed an integrated system, GUITar, to perform primary analysis and hits selection in a user-friendly environment. However, a simple list of hit genes fails to describe accurately enough the complex biological processes that can be triggered by RNAi experiments.

Since a cell function originates from several interactions built among gene networks, sophisticated network-based approaches are needed to decipher these interactions, and understand how they affect the biological system. Indeed, network analyses avoid pre-defined gene sets or pathways and rather identify new pathways that are of interest on the given biological phenomenon from a gene interaction map. It allows the potential discovery of pathways that play a role in the studied cellular function, as the analysis expands from background knowledge to uncharted groups of genes. Integration of gene scores and interaction data also allows to lower the noise, that is, to reduce false-positive/false-negative rates, for instance in the search of a gene signature, as it provides an additional level of validation [7]. In the case of a therapeutic target search, it allows to identify a subnetwork rather that an isolated protein and to be more specific. This kind of approach has been applied successfully using gene expression data for tumor classification [7], [8], prediction of drug response [9], and tumor stratification from variants identified with next-generation sequencing data [10].

Gonzalez & Zimmer [11] were among the pioneers of network-based approaches for RNAi screenings analyses. They developed a method based on co-clustering that was applied to the discovery of host factors of the Hepatitis C Virus (HCV). They identified deregulated modules by calculating a distance matrix among all genes, combining the RNAi score and the shortest path that separates them in an interactome map. They then performed average linkage clustering. Clusters were formed and their size was controlled using an FDR approach. The advantage of their method is that it avoids interactome search heuristics. However, it uses p-values from the RNAi score, which are not always available. Also, there is a difficulty in finding modules of interest in the clustering tree.

Since this study, other algorithms were also proposed for network analysis of RNAi screen [12,13,14,15,16,17,18,19] and recently, two programs were developed, that optimize RNAi screen analysis: HotNet2 and CARD. HotNet2 (HotNet diffusion-oriented subnetworks) [20] is a network analysis program initially designed for discovery of genes and pathways significantly mutated in cancer. The authors performed a pan-cancer analysis using a huge dataset from The Cancer Genome Atlas, and found 16 mutated pathways that comprise known cancer signaling pathways as well as pathways that are less known for their involvement in cancer. This network approach helps identify rare combinations of mutations. HotNet2 uses a heat diffusion process to assess simultaneously the local genes mutations and local topology. HotNet2 starts with initial mutation frequency represented under the form of heat. It then undergoes a heat diffusion process where the heat is diffused among neighbors in the network. An exchanged heat matrix is computed and then so-called ‘hot’ subnetworks are identified. It is also possible to provide the program with a gene score of any type (such as Z-score from RNAi screens) that will be converted into heat and therefore use it for other analysis type. The statistical test provided determines the significance on the combined number and size of subnetworks found.

Dutta [21] proposed CARD, an interactive web-based application dedicated specifically to the analysis of RNAi screenings. It features an extended pipeline, from fully featured data normalization with per-plate quality control, to advanced functional network analysis. CARD features a separate program for each functionality, which potentially allows to process quality control, basic functionalities, and high-level network analyses separately. The program RNAiCut is the first step for network analyses. It combines screening data and PPI data by calculating the probability for an identified hit to have a higher connectivity than what is expected by chance. It helps the user setting a cutoff on z-scores for the gene hits for the following steps of the analysis. The Network analysis of screen hits comes after. The algorithm works by mapping genes into a PPI network (interactome) to identify connections between hits candidates. This step allows the inclusion of connectors to hits, that have a lower z-score but are nevertheless pertinent for the analysis. The CARD application also allows looking for enrichment of screen hits with canonical pathways and GOING terms.

In this paper, we present a new method called HTS-Net, a substantial adaptation and improvement of the Interactome-Transcriptome Integration (ITI) algorithm [7], that is specifically tailored for RNAi screenings network analyses. The ITI algorithm was rewritten in order to optimize the subnetworks detection, by investigating all of the seed's neighboring nodes, rather than extending the subnetworks from one particular neighbor. Moreover, we integrated regulation data into the framework, rather than just investigating PPIs. Indeed, a recurrent limitation of previously cited methods is their sole reliance on PPI data, which is not adapted to understand regulation mechanisms. Our method works by discovering subnetworks with a search heuristics that works in parallel on a PPI network and a regulation network (TF-DNA interaction). In practice, we superimpose RNAi-based gene scores on the interaction and the regulation maps separately. Each one of them is then searched for high scoring areas. The identified regions of interest, the so-called subnetworks, are extracted and reported. We then merge the obtained modules to form meta-subnetworks that incorporate regulation information and PPIs. This approach allows reprioritizing genes by replacing hits into their biological context, which includes their physical interactors and their regulators. We performed our analysis on state-of-the-art PPI and regulation databases, and proved its usefulness on three RNAi screen datasets. In each analysis, we reported newly found markers, GO enrichments and comparison with the original analyses. We also compared our method to other existing algorithms. The program was made available to the community through the HTS-Net (High-Throughput Screening-Network) Mobyle server (http://htsnet.marseille.inserm.fr/).

## Material and methods

The HTS-Net algorithm works as follows. RNAi data is obtained under the form of a list of gene scores (resulting from siRNA assays, shRNAi assays…). Two networks are built, interactome (PPI) and regulome (TF-genes interactions). Gene z-scores are superimposed on each map separately. Then, network regions (subnetworks) with high scores are identified and statistically validated by comparison with a null score distribution. Subnetworks identified in interactome and regulome maps are then merged using common genes, yielding meta-subnetworks with high z-scores.

### Network Data Collection

**Fig 1:**
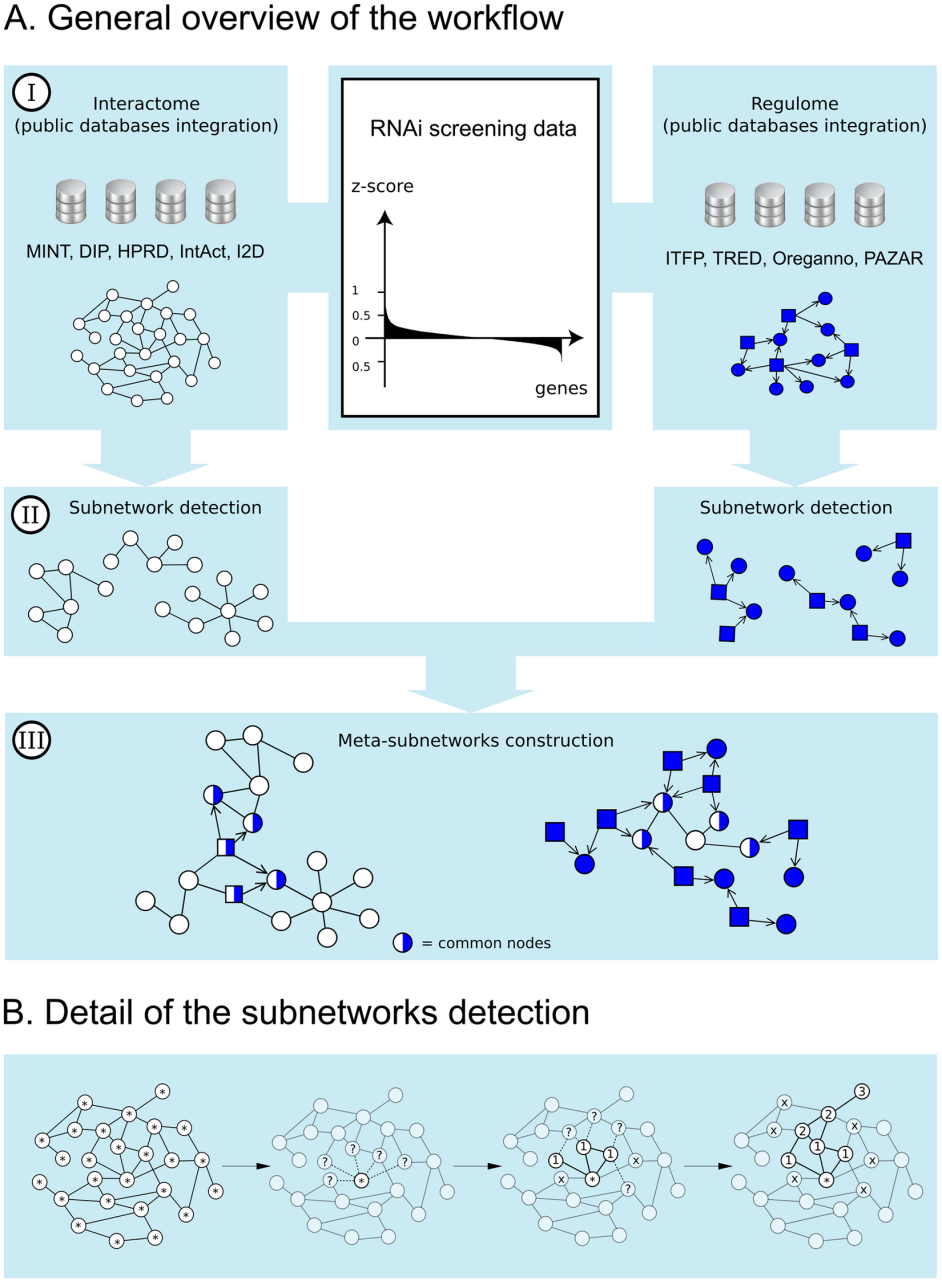
HTS-Net framework. Fig 1A shows the general framework. HTS-Net has been organized in 3 steps of (I) data gathering for the interactome (white) and the regulome (blue) (II) subnetworks detection (performed separately in regulome and interactome) and (III) regulome-interactome subnetworks integration into meta-subnetworks. TF are represented as squares and their interactions are marked as directed. Fig 1B shows details of subnetworks detection. Each node of the network (here, the interactome) is used as a seed for the algorithm. Its neighbors are aggregated if they increase the temporary subnetwork score over a threshold *th*. If they don't, the path is closed. Once all neighbors have been either explored or rejected, the subnetwork is complete.

The discovery of new pathways heavily relies on the coverage, quality, and nature of the network map. We decided to process protein-protein interaction data and TF-gene regulation data separately. Thus, we built two networks by aggregation of several databases: an interactome and a so-called “regulome” (Fig 1).

PPI databases mainly contain data generated by high-throughput methods (Y2H, mass spectrometry, protein microarray) aggregated to “low throughput” data (manually curated data from the literature). In order to build our human interactome, we downloaded, parsed and merged all of the interactions found in major databases: the Database of Interacting Proteins (DIP) [22], the Human Protein Reference Database (HPRD) [23], the Interologous Interaction Database (I2D) [24], IntAct [25], the Molecular INTeraction database (MINT) [26] and Human ProteinPedia [27]. A total of 14,423 genes and 171,146 interactions were gathered in the interactome network.

TF-TF interactions were gathered from TRANSFAC, from the atlas of combinatorial transcriptional regulation by Ravasi and colleagues [28], the interactions predicted by Myšičková and Vingron [29], and those predicted by Yu and colleagues [30]. These were included in the interactome.

Large-scale regulation databases contain predicted human transcription factors (TF) regulation for tissue-specific sets of genes. ITFP (the Integrated Platform of mammalian Transcription Factors) reports 4,105 putative TFs and 69,496 potential TF-target pairs for human [31]. TRED (the Transcriptional Regulatory Elements Database) reports 7,479 targets genes [32]. TRANSFAC, a commercial database, reports 707 TF and 8,712 TF-genes pairs [33]. ORegAnno, the open-access community-driven resource for regulatory annotation, reports 465 TFs and 3,853 genes using a community annotation system [34]. The PAZAR database of gene regulatory information reports 1,284 regulated genes for 708 TFs [35]. A total of 9,755 genes and 68,703 interactions were mapped in the regulome network.

For both networks, all protein identifiers were mapped to Entrez GeneID accession numbers using correspondence tables downloaded from NCBI FTP site (date: November 5th, 2014). The transcription factors from these databases were annotated as such in both the interactome and the regulome.

### Subnetworks detection with HTS-Net

HTS-Net subnetwork scores are formally defined as the average z-score value for all genes belonging to this subnetwork, as follows:

$$S\left(sn\right)=\sum_{g\in sn}z-score\left(g\right)$$

where *sn* is the current subnetwork, *S(sn)* its score, *g* the current gene, *z-score(g)* the z-score corresponding to the current gene. For each studied dataset, the complete set of z-scores measured from the screening was investigated by HTS-Net as shown in Fig 1. In each network (interactome or regulome, Fig 1A) every node is tested as a putative seed (Fig 1B). We decided to use identical algorithm for the two networks. Even if only a part of the regulome is acting as regulators we test all genes, even regulated genes, for potential seeds. This allows the detection of subnetworks with regulated genes having high z-scores, that will include transcription factors that may be characterized by a lower score. On the other hand, this allows the identification of lower-score regulated genes all regulated by a common transcription factor characterized by a high score.

From all seeds, the neighbors are then tested independently. This means that each neighbor of the first layer is tested for score improvement independently and flagged as kept or removed in the current subnetwork. A node is kept if its inclusion allows the increase the subnetwork score over an improvement score threshold *th*.

Then, neighbors that are improving the subnetwork score are added simultaneously and the new subnetwork score is computed. The neighbors of the kept nodes are then explored recursively, layer after layer, in a breadth-first manner. This approach is a strong improvement over the one developed in ITI, which was biased with the order of neighbors’ exploration.

**Fig 2:**
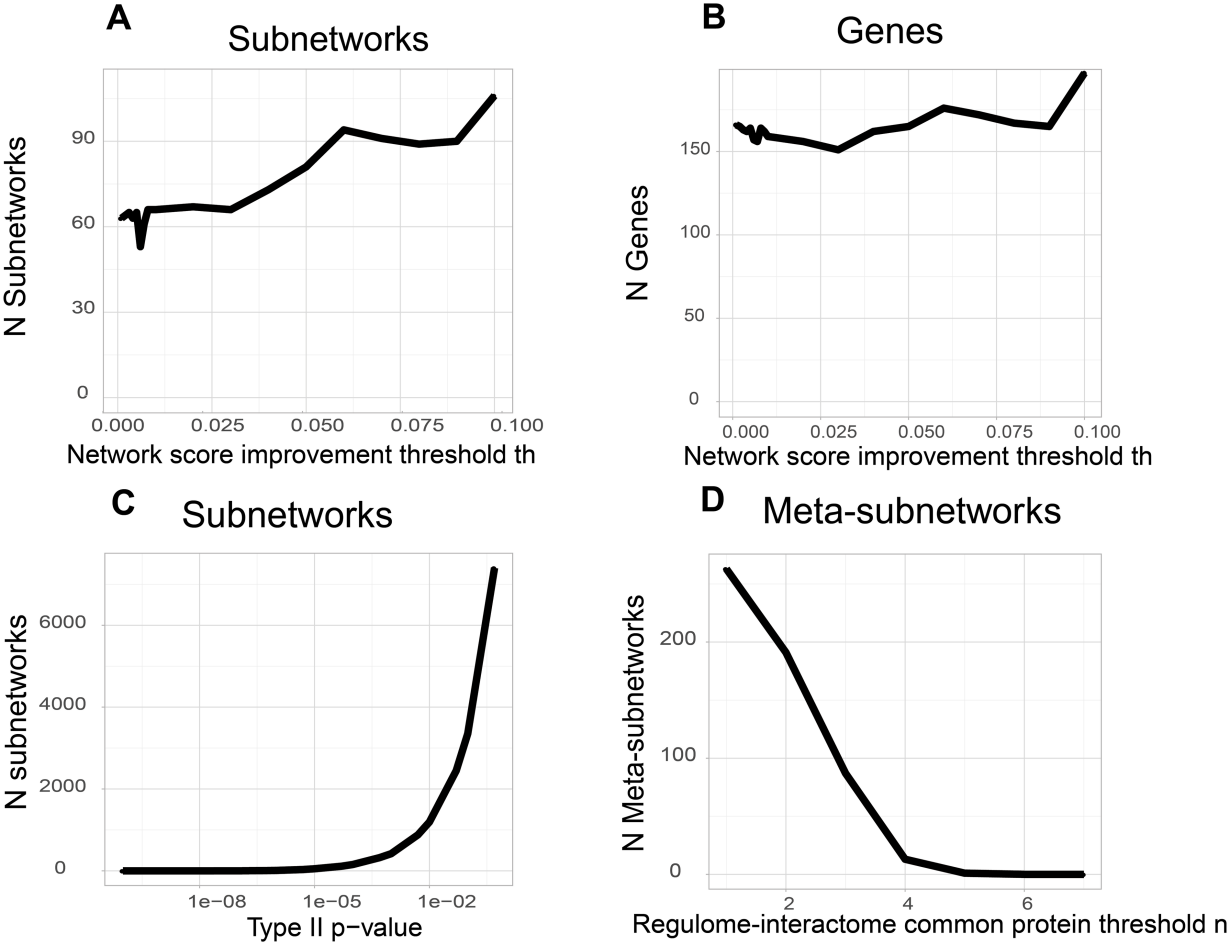
Analysis of HTS-Net parameters impact. We analyzed the impact of several parameters on the number of subnetworks, genes and meta-subnetworks in Chia's dataset (Chia et al., 2010). We tested the impact of minimum score improvement threshold *th* over the number of subnetworks and genes, the impact of a type II p-value on the number of subnetworks, and the impact of the interactome-regulome common protein threshold on the number of meta-subnetworks.

The improvement threshold *th* has a somewhat limited impact on the number of subnetworks and genes (Fig 2). By varying *th* between 0 (less stringent) to 0.1 (more stringent) the number of subnetwork is slightly increased from 60 to 110. This is an opposite behavior to what is intuitively expected. However, a lower *th* value diminishes the overall relevance of the subnetworks produced, which are filtered out later with the statistical validation. The same phenomenon is observed with the genes, which vary from 160 to 190 for a *th* value on the range 0–0,1. For most analyses, we therefore adopted a threshold of 0,05, which is a good compromise between obtaining a number of subnetworks too large for further analysis and a too stringent approach.

In the set of identified subnetworks, there can be high rates of overlap between nodes. It is caused by the fact that every node in the interactome and the regulome is used as a seed by the algorithm: two distant seeds can have a pool of high-scoring genes in common. Before processing with statistical validation, redundant subnetworks should be filtered out, depending on their respective sizes and scores. All the subnetworks are compared by pairs. We compute the proportion of nodes in subnetwork A that are also found in subnetwork B (first overlap), and vice versa (second overlap). If the two overlaps are above a specific rate (80% by default), the subnetwork with a lower score is discarded. If only one overlap is above 80%, it is likely that there is a difference in subnetwork size. If the smaller one has a lower score than the bigger one, it is discarded. If it has a higher score, both subnetworks are kept. We made this choice in order to avoid discarding large subnetworks, and possibly losing relevant information. After this filtering step, the remaining subnetworks are considered for statistical validation.

### Statistical validation

In order to assess the statistical significance of the detected subnetworks, their scores are compared against three distributions of subnetwork scores randomly generated, following the strategy described by Garcia and colleagues (Garcia et al., 2012). The first distribution is obtained by shuffling the scores of the genes resulting from the RNAi screening before detecting the subnetworks. A p-value is then computed by comparing the scores of the detected subnetworks with the scores of this random distribution. This p-value I enforces the link between the RNAi z-scores and the subnetworks, and shows the relevance of associating HTS and network approaches. The p-value II is obtained by detecting subnetworks on randomized network data: all the edges of the networks (interactome and regulome independently) are randomly mixed, keeping only the degree of each node. This second p-value enforces the link between the subnetworks and the global network. The p-value III shows the statistical significance of the algorithm by comparing the subnetworks against randomly generated subnetworks of the same size, regardless of the RNAi scores and network topology.

**Fig 3:**
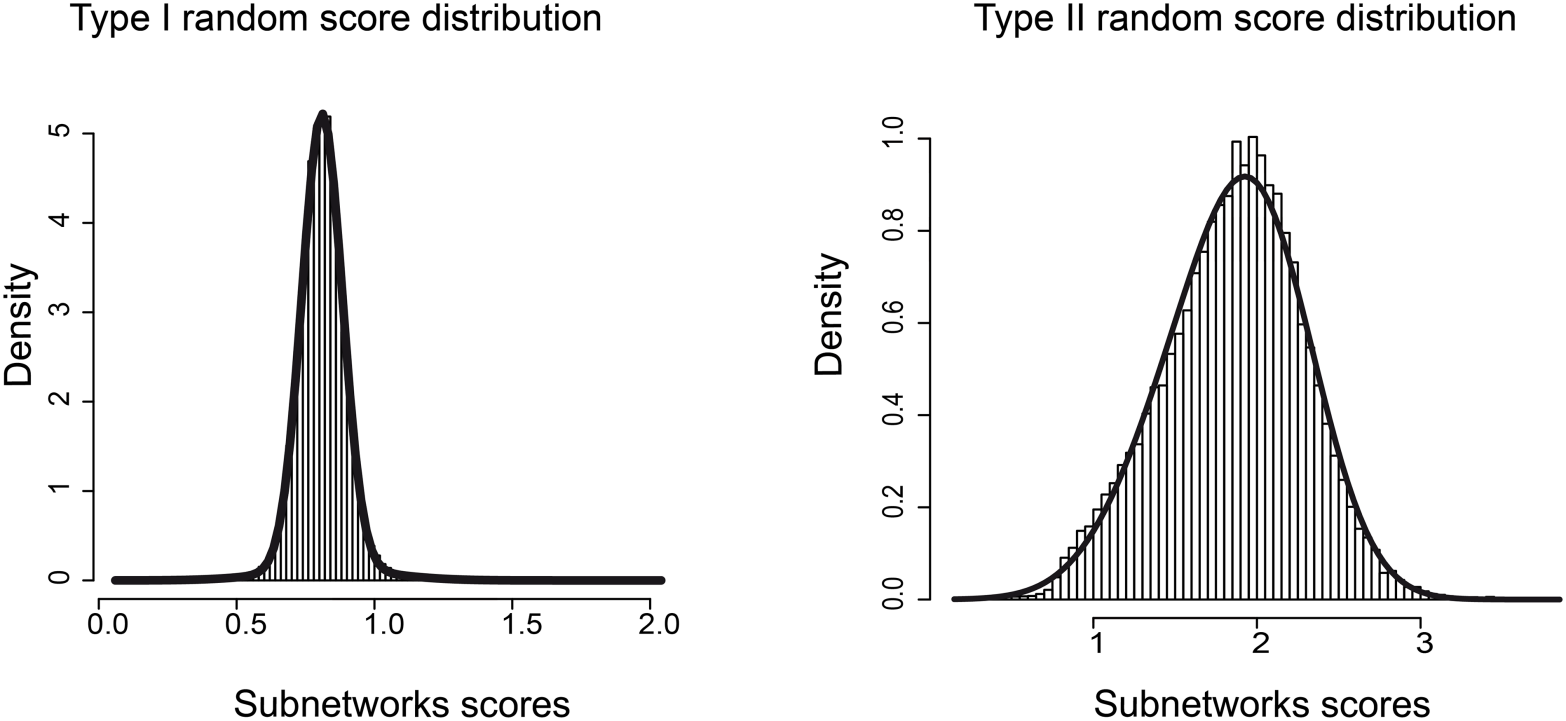
Null score distribution. Examples of Type I and Type II null score distribution obtained with HTS-Net for the Chia dataset (histogram). The distributions are fitted with a Gaussian Mixture model (thick black line).

These three distributions are fitted with a Gaussian mixture distribution (Fig 3) (R package *nor1mix*). The selection of the subnetworks is then made by thresholding all p-values at a statistically significant level, after Bonferroni correction has been applied for multiple testing. In both the interactome and the regulome, final subnetworks are retained if the three p-values are significant. The p-values retained for the three analyses are shown in Table 1. We decided to set these thresholds so that we have a similar order of magnitude for the number of subnetworks to process in each dataset and network. For instance, setting up a p-value of 1e-2 for the dataset from Chia et al. yields 766 subnetworks for the regulome. Fig 2C shows that the number of subnetworks increases exponentially when the p-value II threshold is increased. Similar results are obtained with p-value of type I and III. Setting p-values to a strongly significant 1x10-4 gave us a reasonable amount of subnetworks in the case of Chia's dataset. Differences in subnetworks number are due to differences in datasets topology, and the correlation between HTS z-scores and network. As in any algorithm based on a heuristics, parameters are chosen through analysis of several datasets.

**Table 1:** Choice of the parameters and obtained scores for the three analyses.

Dataset	th	P-value of type I, II and III. (interactome, regulome)	# Subnetworks (interactome, regulome)	Range of interactome scores	Range of regulome scores	*n* (# Meta-subnetworks)
**Chia**	0.05	5x10^-3^; 1x10^-2^	134; 130	2.79; 3.54	2.19; 2.69	4 (12)
**Tai**	0.05	5x10^-3^; 5x10^-3^	32; 28	6.17; 9.06	4.23; 5.67	2 (48)
**Wolf**	0.05	5x10^-2^; 1x10^-2^	178; 54	0.91; 1.15	0.80; 0.97	3 (136)

The columns are describing the following sets of parameters: th is the improvement score threshold. It was set to 0.05 for each dataset. P-values of type I,II and III are described in the 2.4 section. The same value was chosen for the three p-values in the interactome and in the regulome, independently. The range of interactome and regulome scores corresponds to the maximum and minimum subnetwork scores obtained after statistical validation for both networks. n is the threshold of common nodes that was used for combining regulome and interactome networks The corresponding number of meta subnetworks is shown within parenthesis.

### Regulome and interactome subnetworks integration

In order to identify pathways of regulation involving multiple regulation levels (for instance regulome subnetworks regulating several PPI subnetworks), HTS-Net features an integration step to combine interactome-based subnetworks with regulome-based subnetworks by finding common proteins among the two subnetworks types. We examined individually all regulome subnetworks and all interactome subnetworks and connected subnetworks that presented a minimum of *n* common nodes, yielding meta-subnetworks (Fig 1). This value depends on the convergence of subnetworks and is dataset-specific.

We studied the variation of the number of obtained meta-subnetworks versus the common protein threshold (Fig 2D). The number of meta-subnetworks is greatly affected by the number of common proteins *n* between regulome and interactome, from around 200 meta-subnetworks obtained for 1 common protein to 0 for 6 common proteins. This reveals the modular nature of regulome and interactome subnetworks which are linked by a limited number of proteins. For Chia’s dataset, we observe a break in the curve decrease around 4 proteins, which gave a manageable number of 12 meta-subnetworks.

Table 1 gives an overview of all the parameters chosen for each analysis.

### Online reports and subnetworks databases

The heterogeneous nature of data sources, the complex conversion of gene IDs and the overall structure complexity of the newly generated data necessitate an advanced visualization system in order to properly interpret the nature of subnetworks. In particular, several interaction types as well as several network types (interactome, regulome) must be accordingly visualized, and compared to known biology. The HTS-Net screening pipeline features an advanced reporting mechanisms allowing subnetworks visualization while taking into account several network types and various interaction types (PPI, gene regulation).

All detected subnetworks are plotted using GraphViz package (AT&T Research) and the web report design was done with KickStart 0.99 (HTML5+CSS3). Using this interface, all subnetworks can be investigated and compared. All genes are also listed and connected to their respective subnetworks. NCBI links and GO enrichment analyses are provided. Further analysis with Cytoscape is straightforward, as all subnetworks are generated using the standard Network Nested File format. All the data is available as a flat file for further analysis from the HTS-Net companion web site.

### GO enrichment statistics

Genes were annotated with Gene Ontology (GO). GO enrichment analysis was performed for each dataset by standard hypergeometric testing associated with a Benjamini-Hotchberg multiple testing correction (FDR = 5%) [36]. Gene Ontology enrichment of all subnetworks for the three tested datasets are available as an Excel file made available as S1 File and from the HTS-Net web site (http://htsnet.marseille.inserm.fr/supplementary-data.html). Results from the GO enrichment analysis are discussed in the results section for the three analyzed datasets.

### Public data sources

In order to evaluate the capacity of our HTS-Net pipeline to identify gene networks of interest, we reanalyzed three published RNAi screenings datasets: the dataset published by Chia and colleagues [37], the dataset published by Wolf and colleagues [38], and the dataset published by Tai and colleagues [39].

Chia and colleagues [37] set up an RNAi screen to determine genes that sign for human embryonic stem cell (hESC) identity. First, they engineered a cell line model, H1 hESC [40], by introducing a GFP reporter construct driven by the POU5F1 upstream regulatory region. POU5F1 expression can be considered as a marker of undifferentiated hESC. This cell line model was screened against a library of 21,212 human genes in duplicate. The mean of the two z-scores for GFP fluorescence reduction (Fav) and Nuclei number reduction (Nav) were calculated. The authors found all genes with a Fav_score >2 to be potential candidate determinants of hESC identity, which gave a total of 566 genes. They then performed a secondary screening on the 200 most highly ranked genes.

The second dataset reanalyzed concerns a study by Wolf and colleagues [38] where they tested the ability of breast cancer stem cell (CSC) population (CD44+CD24-/low) to form mammospheres (a functional surrogate marker of the CSC self-renewal ability) after short-hairpin RNA interference assay (shRNAi) to identify breast CSC key regulators on 5,045 genes. They compared this analysis with the same cells cultured in adherent conditions (culture condition allowing cell differentiation) to eliminate shRNAi without breast CSC-specific effect. Wolf et al. initially selected 1,051 genes with a p-value <0.01 on the adherent z-score to assess genes impairing cell growth. Then they performed an enrichment analysis on a set of 392 genes specific to mammosphere formation, to verify a p-value <0.01 on the mammosphere z-score and a p-value >0.1 on the adherent z-score to enforce CSC specificity. They then focused the analysis on autophagy genes ATG4A and ATG4B, with respective z-scores of 0.51 and 0.11.

The last dataset reanalyzed is the one from Tai et al. [39], who screened for host proteins involved in HCV replication using the Huh7/Rep-Feo subgenomic genotype replicon cell line. This replicon encodes the HCV non-structural proteins that mediate viral transcription as well as a firefly fusion protein used for signal quantification. This cell line was screened against 21,094 siRNAs targeting the whole human genome in duplicate.

**Fig 4:**
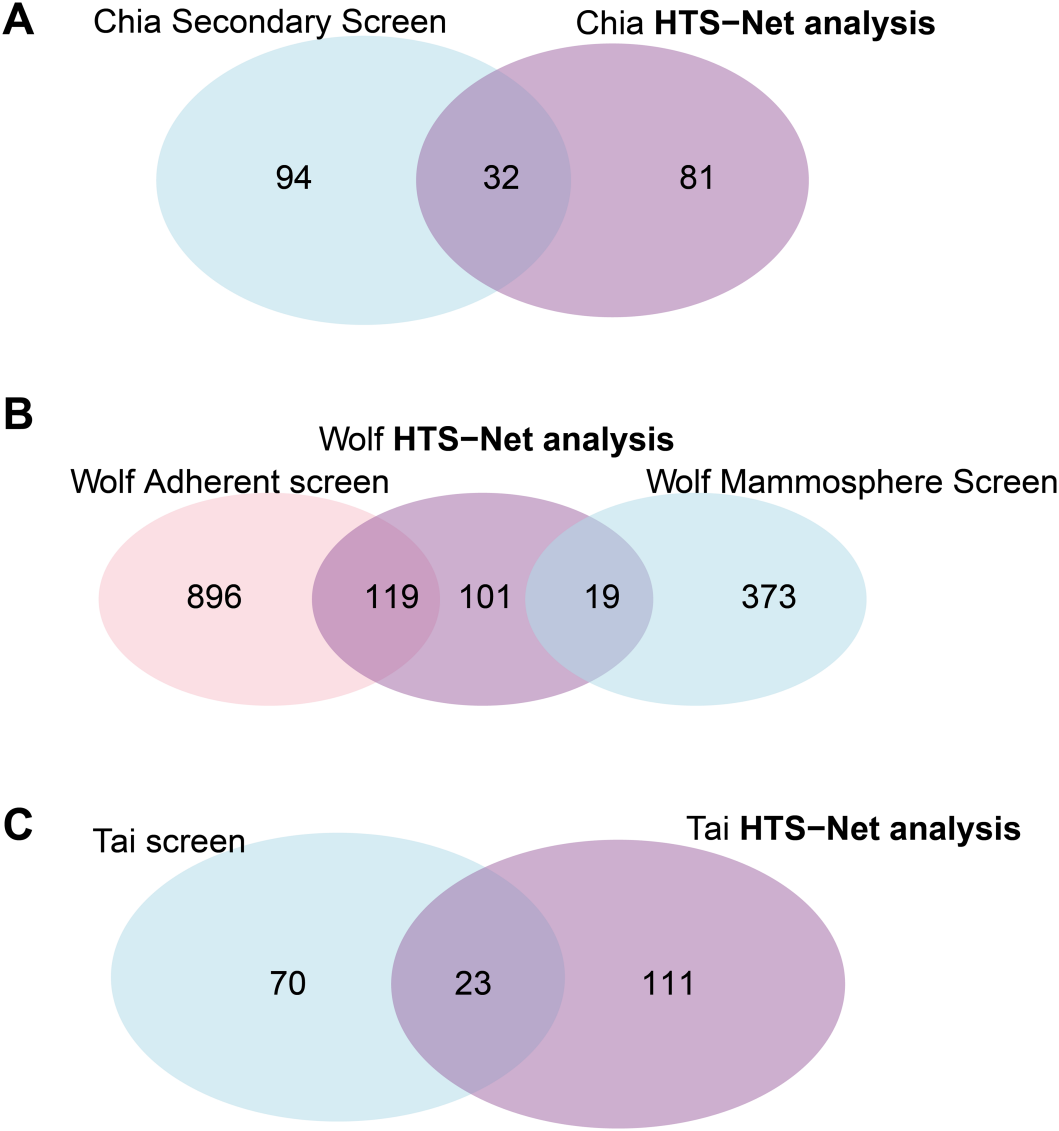
Overlaps of genes between different algorithms and HTS-Net. The figure shows Venn diagrams of the different overlaps obtained between HTS-Net runs on Chia, Wolf and Tai datasets and runs of other algorithms by the original authors. The A Venn diagram shows the overlap between the Chia secondary screen and the HTS-Net analysis. The B Venn diagram shows the overlap between the Wolf Adherent-enriched screen, the Wolf Mammosphere enriched screen and the corresponding HTS-Net analysis. The C Venn diagram shows the overlap between the Tai original screen and the HTS-Net analysis.

The web-based report (companion web site) shows how the significant genes and their associated networks stand among the genes and z-scores previously detected by the original analyses. Fig 4 shows overlap (Venn diagrams) between the original analyses and genes identified with HTS-Net and are commented in the Results section.

### Comparison with Hotnet2 and CARD programs on the Tai dataset

We compared HTS-Net with three other programs, Hotnet2, CARD and Gonzales & Zimmers’s method on Tai’s dataset. The choice for the Tai’s dataset was motivated by the fact that Gonzales & Zimmer do not provide source code or program but published an analysis of Tai’s dataset in their publication, so this was a good basis of comparison. Hotnet2 is provided under source code. We therefore installed the code in a Linux machine, and compiled it after installing along all dependencies. We then ran it with the HPRD [23] network after generating corresponding influences matrices for the networks (matrices that measures how the heat will propagate within the network). The HPRD network was chosen since it is distributed along with the program source code and is well integrated with it. The program runs with two parameters, Beta and Delta. Beta is a network heat diffusion parameter that sets how a node value interferes with its neighbors. We left it at its default value. Delta is the edge weight and has an impact on the detected subnetwork sizes. After running, the program proposes several values on the range [2,45x10^-3^–4,92x10^-3^] and we chose Delta = 2,44x10^-3^ which gave reasonable subnetworks number and size (288 subnetworks, p = 0.46). We used the subnetwork export function to obtain a gene list comparable with other pipelines.

CARD being accessible as a web service, we registered an account and formatted Tai's data to the CARD Format. Chrome 44 is officially recommended, but we were able to run it with the more recent version 55. There was no way to directly provide normalized RNAi data, so we provided randomized identifiers for plate, as this information was not available for the dataset analyzed, and loaded the data by specifying no normalization, so that we kept the previously normalized data. Then, we identified hits using RNAiCut to determine optimal level of significance for z-score. The maximum log(p-value) occurs at 2,786 (p-value = 0,004).

We then started the network analysis with this setting. We selected all networks available in the system, HPRD, Bind and BioGRID. We downloaded the resulting gene list as a CSV file in order to compare results with other pipelines.

For Gonzales & Zimmer’s method, the code was not available, so we re-used the analysis of Tai data provided in the corresponding article.

## Results

### A new pipeline for deciphering regulation and interactions impacted by HT screens

We are proposing the HTS-Net pipeline for the network analysis of large-scale (up to genome-wide) RNAi screenings adapted to various experimental settings. The pipeline is maintained and accessible through a locally-deployed Mobyle portal [41] available at http://htsnet.marseille.inserm.fr/. A tutorial is available from the same URL. Analyses can be conducted as soon as the data is normalized and properly formatted to HTS-Net, under the form of a 2-column tab-delimited file: the first column contains gene identifiers (Entrez Gene), and the second column contains associated z-scores from the screening. If multiple replicates exist, they must be averaged or combined to a single score, as HTS-Net is not designed to work with multiple scores simultaneously. Additional information must be provided, that is, the organism studied and the p-values for scoring. An archive is then generated that contains the analysis report in HTML format compatible with any standard web browser.

In order to understand the results obtained from the HTS-Net pipeline, we compared genes identified by HTS-Net with genes originally published. We also produced GO enrichment analysis for genes that are in common between HTS-Net and the original publication and genes that have been specifically identified with one of the two methods (see support data on companion web site).

**Fig 5:**
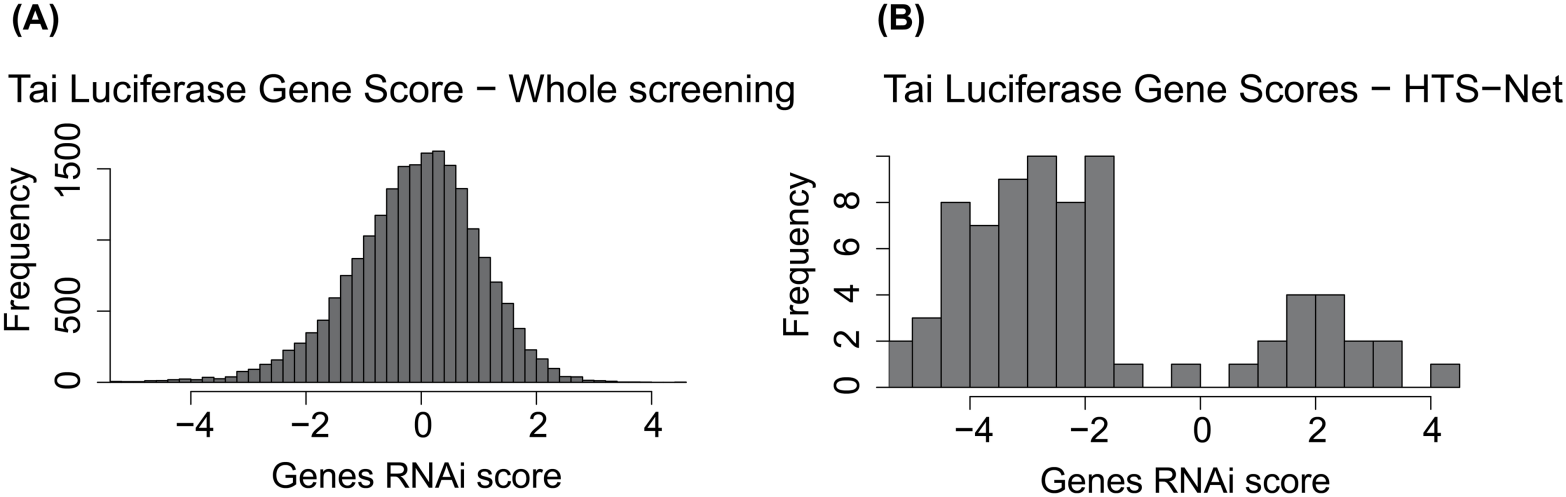
Z-Score distribution of RNAi screening for the Tai dataset (A) versus distribution of genes detected by HTS-Net (B). This plot shows that several genes are retained by HTS-Net are below the score retained by typical analysis.

Fig 5 shows the distribution of z-scores for the Tai Luciferase dataset along the distribution of score for genes identified with HTS-Net. These distributions show that HTS-Net is less stringent with genes that have a low score but connected to important networks, and that would have been discarded by a classical analysis based solely on z-score statistics.

### Network analysis of human embryonic cell differentiation determinants (Chia et al.)

We pursued the analysis made by Chia et al. with the Fav value, as in the original paper, and fed it to the HTS-Net pipeline as a gene score. The minimal score increase threshold *th* was fixed to 0.05. 21,023 genes were mapped to the interactome and the regulome, in order to detect subnetworks playing a role in differentiation. We performed an HTS-Net analysis with a p-value cutoff of 5x10^-3^ for the interactome analysis and a p-value cutoff of 1x10^-2^ or the regulome analysis in order to obtain comparable number of subnetworks for the two networks while keeping a statistically significant score. HTS-Net was set up to detect genes with highly negative z-scores, as in the original publication. We respectively obtained 134 interactome and 130 regulome subnetworks that were integrated into 12 meta-subnetworks after performing integration on a threshold of 4 common nodes. These were comprised of 294 genes for the interactome, 330 genes for the regulome, and 113 genes after identification of meta-subnetworks. There are 32 genes found common between the 113 genes identified by HTS-Net and the 126 genes identified by Chia (Fig 4A).

Chia focused on transcriptional regulators PRDM14, NFRKB and YAP1. All were found present in high-scoring subnetworks. PRDM14 was found in 7 regulome subnetworks. The base regulome subnetwork related to PRDM14 has a score of 2.412. NFRKB was detected in 48 interactome subnetworks, 3 regulome subnetworks and retained in 3 meta-subnetworks. The best NFRKB-related interactome subnetwork score was 3.11. YAP1 was detected in 94 interactome subnetworks and 12 meta-subnetworks. The best YAP1-related subnetwork score was 3.54.

**Fig 6:**
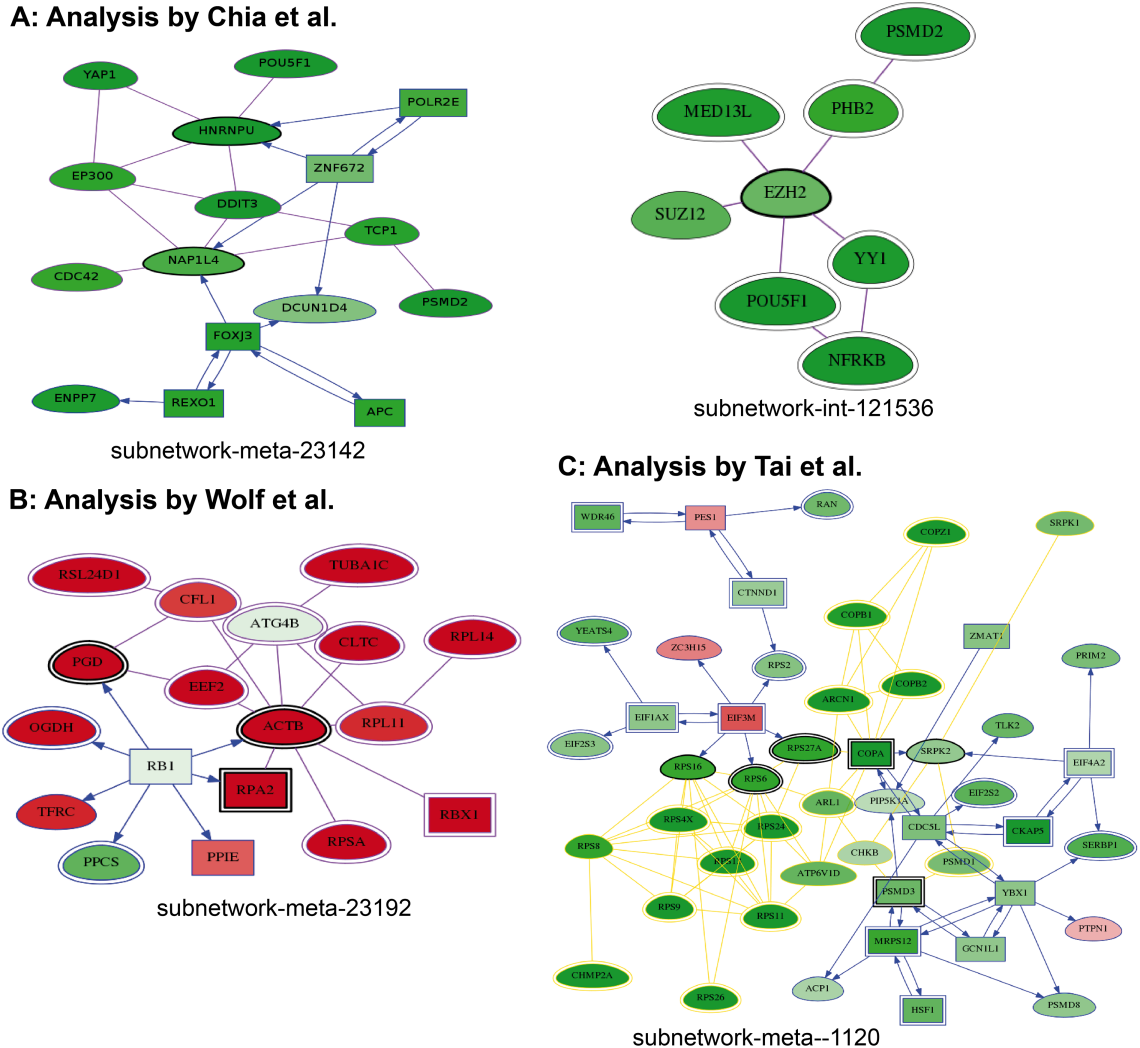
Examples of networks detected in each analysis. 6A: Chia analysis. EZH2 deregulated interactome subnetworks are shown. Darker green correspond to higher z-score. 6B: Wolf analysis. The Meta subnetwork related to ATG4B is shown. Red = more elevated in mammosphere (meaning silenced in adherent), green = more elevated in Adherent (meaning silenced in mammosphere, as expected for ATG4B). 6C: Tai analysis showing the interactions of PIP5K1A including OPA, PSMD1 and the ribosomal machinery (red = negative z-score corresponding to HCV machinery inhibition, green = positive z-score).

Thanks to the HTS-Net pipeline, we were able to identify other interesting genes from this dataset. Several genes from the Polycomb Repressive Complex 2 (PRC2) were highly ranked by HTS-Net but left apart by Chia’s method. These chromatin regulators are known best for their function in establishing and maintaining epigenetic memory during development. The gene EZH2, known for playing a role in embryonic stem cell self-renewal by maintaining cellular identity [42], has a screening score of -1.631 and was not retained by Chia. It was detected by HTS-Net to be present in 2 interactome subnetworks and 5 regulome subnetworks. It was found in subnetworks int-snw-2146 as the seed, interacting with another PRC2 member, SUZ12, and with a number of other genes detected by Chia and colleagues (Fig 6A). SUZ12 is involved in cellular differentiation and has been found involved in the progression of a number of cancers [43]. It was also found in subnetwork int-snw-121536 with AEBP2 as the seed, also having a score lower than Chia’s cutoff. AEBP2 is required for optimal enzymatic activity of the PRC2 complex [44]. In subnetwork int-snw-54857, several genes identified by Chia are present (YAP1; NFRKB) but the subnetwork seed GDPD2 and the gene HEXDC were specifically identified by HTS-Net. GDPD2 codes for an enzyme that may play a role in osteoblast differentiation [45]. Parameters for HTS-Net analyses reported in this paper were summarized in Table 1.

The GO enrichment analysis (see supplementary data) for genes found with HTS-Net and genes found in Chia’s secondary screening showed several GO terms having a specific enrichment found only in HTS-Net analysis (See supplementary GO table on the HTS-Net web site). General processes were detected by both approaches, such as ‘gene expression’ and ‘protein binding’. However, specific processes were detected as highly significant by HTS-Net, such as ‘viral process’ (16 genes– 9 found by Chia et al.). Genes located in the cytosol (38 genes– 26 found by Chia et al.) are also more enriched. HTS-Net identified ‘Cop9 signalosome’ (6 genes, 2 by Chia et al), as well as genes from the mediator complex (5 genes, and none by Chia et al.). HTS-Net also identified ‘poly(A) RNA binding’ (27 genes—only 17 found by Chia et al.). Other specific enrichments found in Chia’s secondary screening were detected at the limit of significance at FDR 0.05 by HTS-Net, such as ‘oligodendrocyte development’, ‘cellular response to stimulus’ and processes related to ‘negative regulation of transcription’ and ‘negative regulation of translational initiation in response to stress’.

### Network analysis of mammosphere formation (Wolf et al.)

Wolf’s shRNAi assay was performed on 5,045 genes that we reanalyzed with HTS-Net. These genes reported in the supplementary material were converted to 4,948 Entrez Gene identifiers to ensure HTS-Net compatibility.

We setup an integrated HTS-Net analysis with both the mammosphere-enriched and the adherent-enriched screening data. In order to select subnetworks differentially affected in the two conditions, we computed the ratios mammosphere/adherent for each gene knockdown, and fed it to HTS-Net with a improvement threshold th = 0.05 and p-values of 0.05 for the interactome search and 0.01 for the regulome search. Absolute value for ratio is used in order to detect both mammosphere-specific networks and adherent-specific networks in a single HTS-Net run.

The HTS-Net approach identified 239 genes, organized in 136 meta-subneworks, 178 interactome subnetworks and 54 regulome subnetworks, and allowed an integrated analysis of mammosphere-enriched cells and adherent cells. The Venn diagram (Fig 4B) reveals that we mainly detected adherent-specific genes, as the adherent z-score tends to be higher than the mammosphere z-score.

The ATG4A gene was not detected by HTS-Net, but ATG4B was detected as seed of a subnetwork globally over-expressed in mammosphere-enriched cells (int-snw-23192, score = 0.993), while ATG4B was itself under-expressed. Importantly, if ATG4B has a quite large degree (number of connected nodes) of 159 in our interactome, it has been specifically added in a single subnetwork. Interactome subnetwork int-snw-23192 was connected to regulome subnetwork reg-snw-2958 to form a regulated structure found in meta-subnetwork meta-reg-snw-23192 (Fig 6B) and a larger structure, meta-reg-snw-2958, composed of 31 subnetworks. Meta-reg-snw-23192 contains 18 genes including TP53, a number of genes modulating microtubules (TUBA1C and ACTB), and genes involved in elongation (EEF2). Other proteins involved in ribosomes are present in this subnetwork.

We found that a large number of the 239 genes detected by HTS-Net have been previously detected as adherent, while only five were identified in mammosphere-enriched cells. This observation is due to the fact that we set up our HTS-Net analysis to detect larger log2(mammosphere/adherent) ratios in absolute value, and that a larger number of genes have a higher expression for adherent cells screen. When analyzing GO terms enrichment, we found that a number of other functions related to cell cycle and cell differentiation were also detected specifically by HTS-Net, for instance, the GO term ‘mitotic cell cycle’ (present in 32/415 genes detected by HTS-Net and 15/515 genes in original publication), the GO term ‘DNA damage response, signal transduction by p53 class mediator resulting in cell cycle arrest’ (13/68 genes with HTS-Net, while 1/68 genes in original publication).

### Network analysis of HCV host cofactor replication (Tai et al.)

Two analyses were previously performed with this dataset: the original from Tai and colleagues and a network-based analysis from Gonzalez & Zimmer [11], which gives the opportunity to make comparisons and understand advantages and limitations of each approach. We performed an analysis with HTS-Net, as reported in this section.

HTS-Net mapped 17,695 knockdowns to Entrez Gene identifiers, 12,906 genes to the interactome and 8,990 genes to the regulome. 141 genes were retained as significant both in interactome and regulome networks and were found in respectively 32 and 28 corresponding subnetworks, and 55 meta-subnetworks. Of the 141 genes identified with HTS-Net, 23 were common with the set published by Tai.

All studies identified genes in the COPI coat. These ranked high in the screening and were also favored in the clustering analysis. We identified a number of genes that are part of this complex with HTS-Net, including COPA, COPB1, COPB2, COPG1, and COPZ1. COPI coat activity was confirmed by Tai and colleagues to be required for HCV replication. CDC42, highly ranked in the screening, was also detected with HTS-Net, which binds to coatomer. Tai and Gonzalez also reported CDC42.

Some genes were reported specifically by HTS-Net. Among them, we found BRCA2, transcription factor known for its role in genome stability and which acts as a predisposition gene in breast and ovarian cancers. A large number of genes related to ribosomal machinery are also reported by HTS-Net (e.g. RPS11, RPS13 and others).

We focused the analysis on meta-reg-snw-7133. This meta-subnetwork resulted from the aggregation of 10 subnetworks and contains a number of regulatory elements. Its center is materialized by interactome subnetwork int-snw-7133 itself regulated by EIF3M, present in three regulome subnetworks. This transcription factor is required for protein synthesis but is also known to be a homolog of Tango7 in drosophila, implicated in Golgi transport [46]. While having a score relatively high (4.41), these genes were not originally detected by Tai et al. as top-scoring genes. As a TF, this could represent a valuable HCV target.

Tai did not initially retain PIP5K1A, which has a mildly significant z-score (-1.820). Nevertheless, it belongs to several high-scoring meta-subnetworks (best meta subnetwork score = 4.751). We found this kinase in a single interactome subnetwork, interacting with COPA, PSMD1 and the ribosomal machinery (Fig 6C) and four meta-subnetworks.

HACD3 (PTPLAD1, BIND1) was also specifically found by HTS-Net. It is a regulator of the viral replication [47]. Tai did not identify this gene because of its lower score (0.875), but its mechanism of action has been shown with HTS-Net. This gene is present in two meta-subnetworks through its presence as the seed of a regulatory subnetwork (reg-snw-51495), which has a high score (4.504).

We also looked for GO enrichment of HTS-Net subnetworks. Among them, int-snw-4381 is significantly enriched for the GO terms ‘viral transcription’ (p = 2.07x10^-27^) and ‘viral life cycle’ (p = 2.95x10^-27^) genes. GO enrichment of all genes identified with HTS-Net is provided as supplementary material.

### Comparison of HTS-Net with existing algorithms

**Fig 7:**
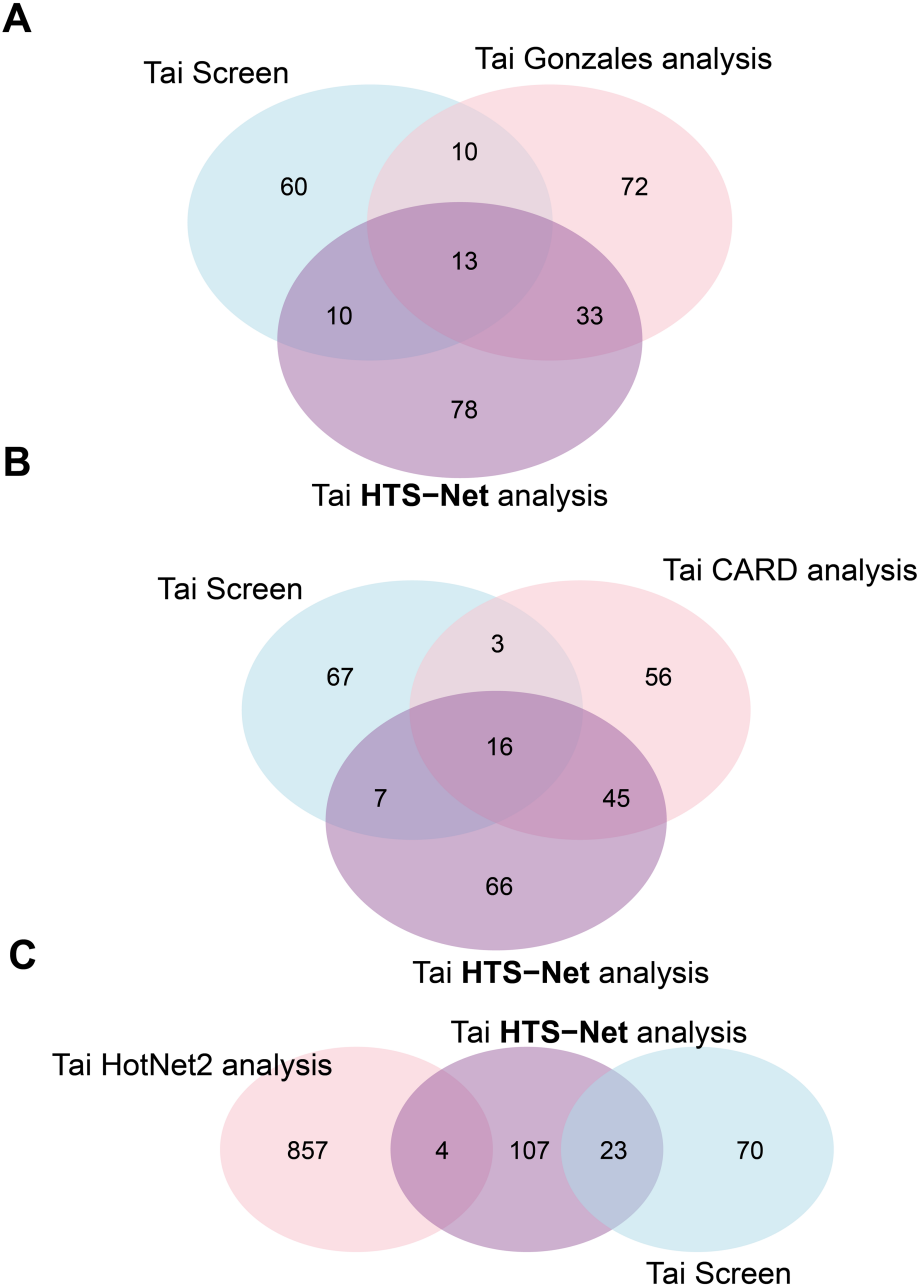
Measure of overlaps between Tai, HTS-Net and other methods. Overlaps between Tai’s initially detected hits, HTS-Net analysis, and other methods are represented under the form of Venn diagrams. In Fig 7A is shown the overlap with Gonzales & Zimmer’s method, in Fig 7B is shown the overlap with CARD and in Fig 7C is shown the overlap with HotNet2.

In order to understand the differences and common points of HTS-Net with existing approaches, we performed a complete analysis of the Tai dataset with three approaches: analysis program by Gonzales & Zimmer [11], specifically designed for RNAi, the general-purpose network analysis program HotNet2 [20] and the CARD suite [21]. We specifically used the Tai dataset since the analysis by Gonzales & Zimmer’s method was only available for this dataset. Lists of hits identified by each method are provided as supplementary material Excel file on the HTS-Net Web site and as S2 File. Key results of the comparison are reported in Table 2. Venn diagrams comparing the overlap among methods are shown Fig 7.

**Table 2:** Comparison between HTS-Net and Gonzales & Zimmer’s method, CARD and HotNet2.

Method	Type	Interactome	Statistical validation	Cost value/subnetwork score	Specificities	Disk footprint	Running time with default parameters.
**HTS-Net**	Web server+Source code executable	HPRD, BIND, DIP, I2D,IntAct, MINT, ProteinPedia, TRED, ITFP, TRANSFAC (internal use only), OregAnno, PAZAAR	Topology of gene network	Maximisation of subnetwork score	Triple statistical validation, regulation analysis on regulome	500Mo	1h48
**Gonzales & Zimmer**	Not provided	STRING	Co clustering on network proximity and score	Maximisation of subnetwork score	Approach based on clustering	Unknown	Unknown
**CARD**	Web Server	HPRD, BIND, BioGrid	Number and size of subnetworks	Gene connectivity	Complete web server that includes low-level RNAi data processing	Unknown	40minutes
**HotNet2**	Source code to be installed	HPRD, iRefIndex	Number and size of subnetworks	Diffusion and maximization of subnetwork score	Initially designed for mutation analysis.	60Go	40+ hours

This table summarizes scores and statistical testing method provided that details are found in each method’s original publication.

#### HTS-Net

The running time was 20 minutes when running the parallelized code version on our local cluster. The public web site run is 1h48 minutes.

HTS-Net allowed the identification of PIP5K1A as hit. Neither Tai nor Gonzalez found this gene in their analyses. Interestingly, it was previously found to be implicated in HCV virus host entry and replication [48]. Other specific findings are detailed section 3.4.

#### Gonzales & Zimmer

Gonzales & Zimmer’s method yielded 128 genes. The number of clusters is not described, but the authors merged them all in one single network. The calculating cost was not specified. Gonzales detected 23 genes initially found by Tai, while 46 genes were also detected by HTS-Net (Fig 7A), showing a closer topology with HTS-Net had also 23 genes common with the set published by Tai. EIF3M has a score relatively high (4.41), but was not originally identified as hits by Tai et al.; it was identified by Gonzalez and HTS-Net. As a TF, this could represent a valuable HCV target.

#### CARD

Using the RNAiCut and Network analysis modules from CARD, we obtained 119 genes (on 3 subnetworks) for network of hits genes (score greater that 2.786) and 493 genes with non hits (z-score lower than 1.0). The system allows downloading networks of hits. We then proceeded with the functional analysis of the obtained genes for comparison with other methods. The running time was about 40 minutes for RNAiCut and a few seconds for Network analysis.

The use of CARD on the Tai dataset revealed 19 genes already detected by Tai, and 61 genes were common with HTS-Net result (Fig 7B). Among them was found NCOA6, a transcriptional coactivator that can interact with nuclear hormone receptors to enhance their transcriptional activator functions. COPA, COPB1, COPB2, COPZ1, the coatomer protein complex subunit alpha, beta 1, beta 2 and zeta1 were identified by CARD. Tai initially detected them as primary hits, and their important role for protein trafficking was detailed in their publication. However, HTS-Net and Gonzales’s method were also able to detect these genes of importance. SERBP1 was identified, also identified by HTS-Net, together with ribosomal proteins and EIF3M, known to interact with the hepatitis C virus internal ribosomal entry site (IRES) [49]. RAN (RAS-related nuclear protein) was detected by all methods with the exception of HotNet2. It is a small GTP binding protein belonging to the RAS superfamily that is essential for the translocation of RNA and proteins through the nuclear pore complex and known to be an HCV antibody [50].

#### HotNet2

We performed a run after establishing influences matrices for HPRD networks plus 100 random influences matrices for statistical analysis, and computing heat matrix for the Tai dataset after appropriate data formatting. After run, HotNet2 returned 4 analyses with 4 different Delta values. Changing Delta had a large impact on the size and number of the subnetworks found. In order to obtain a reasonable subnetwork size, we chose delta = 0,0244, that yielded 36 subnetworks of size 5 or more. The running time was 40h 45min to generate influence matrices for HPRD network (this step is done only once) and 12min 32sec for the HotNet2 run itself, using 4 cores.

There were only 4 genes in common between Hotnet2 and HTS-Net, while no genes initially detected by Tai (Fig 7C). This can be due to the different metric, which was initially designed for mutations and that gives higher weight to network connections with the heat diffusion process. Also, the program only included HPRD in its current distribution, which may be another limiting factor. HotNet2 makes possible to include new interactomes, but the computational cost of calculating influence matrices is prohibitive on our local setting.

We examined results for the top-scoring subnetwork. It contains PPP1CB, which is an interesting hit, although not found by other programs, since it has been found in the first 40 up-regulated genes in HCV-related Hepatocellular carcinoma (HCC) samples analyzed by De Giorgi and colleagues [51]. However, none of the strong hits initially identified by Tai were found.

## Discussion

We developed a new tool, HTS-Net, which uses a systems biology algorithm for the unbiased identification of potential candidates in RNAi screenings. HTS-Net replaces genes in their interaction and regulatory context to discover modules of interacting genes that are biologically relevant to the experiment. We demonstrated the usefulness of our approach on three biological applications: regulation of human embryonic stem cells (Chia et al., 2010)[39] differentiation of breast cancer stem cells [38], and identification of human host-HCV regulators (Tai et al., 2009). [37]

Chia et al. built an RNAi screen for the identification of hESC identity. Our approach allowed identifying a number of genes that were not previously found by Chia. Among them, we identified PRC2 as master regulator of hESC differentiation.

Wolf et al. built an RNAi screening to identify key regulators of breast cancer stem cells (CSC) behavior. The re-analysis with HTS-Net allowed identifying the role of the autophagy regulator ATGA4 in the maintenance of the CSC subpopulation. While HTS-Net did not identify ATG4A to have a role in CSC behavior, we were able to identify ATG4B and its interacting neighbors.

The Tai analysis dealt with the identification of human host proteins that interact with and support HCV replication. We compared results of the original publication and a re-analysis of these data by Gonzalez. All studies identified genes in the COPI coat, but our method was specifically able to identify kinases such as PIP5K1A.

The comparison with other reference programs on the Tai dataset shows that HTS-Net was able to retrieve a large number of hits identified in the original publication while identifying new hits not identified by other network approaches. HTS-Net offers a more complete biological environment for RNAi hits by using regulatory data. To some extent, HTS-Net was closer to other network approaches than Tai (larger overlap). This is due to general similarities in network analysis that are able to include hits having lower scores but higher connectivities. HTS-Net had closer results with CARD and Gonzales & Zimmer, while HotNet2 gave distant results, which may be due to the fact that this is not a method specifically designed for RNAi analysis. In terms of hardware, HTS-Net is provided under source code and cluster implementation, or can be used as a web service, requiring no installation but with a longer running time. CARD is a web-only service, while HotNet2 requires local installation. Gonzales & Zimmer did not provide web service or source code.

## Conclusion

We are proposing the pipeline HTS-Net, a network-based RNAi analysis pipeline, for the analysis of RNAi screening data. The pipeline is available from our Mobyle server, and was tested with three datasets and compared with other networks-based approaches, some specifically designed for RNAi screening analyses (CARD) [21], Gonzales & Zimmer’s approach [11], and other for general-purpose analysis (HotNet2) [20].

Our approach showed many advantages when compared with other methods. It has similar (Gonzales) or more (CARD, Hotnet2) genes found in common with the Tai analysis, which means that fundamental biological information is retrieved, while keeping the ability of discovering new hits of interest. The production of exhaustive reports allows causal exploration of gene networks, since regulatory data is included in the pipeline, feature not proposed by other software (with the exception of HotNet2). Running time stays reasonable on a web server, and fast execution can be provided when needed by local installation and run on a Linux server or cluster.

The HTS-Net algorithm avoids defining a hard cutoff on individual genes, allows bypassing the limitations of traditional analyses and unravels key components for the studied systems, such as master genes regulators. Our approach is a first answer to deciphering the regulatory complexity of studied systems in RNAi screenings. Possible improvements include taking into account the interaction quality when building subnetworks, by introducing weighted vertices reflecting the nature of the interactions and the biological evidence that supports it. As genome-wide experimental setups such as the one presented here are becoming commonplace, tools such as HTS-Net will be more and more useful to carry advanced analytics.

## Supporting information

S1 File Gene Ontology terms enrichment table for HTS-Net runs for Wang, Tai and Wolf datasets. This file contains Gene Ontology terms enrichment information for the 3 HTS-Net runs studied in this paper (Wang, Tai and Wolf datasets) along with count of terms and statistics. Significant GO terms enrichment (using hypergeometric statistical test along a Benjamini-Hotchberg correction fixed at an FDR of 5%) are highlighted in yellow.
(XLSX)

S2 File Genes reported as targets for the Tai dataset. This file lists genes detected as targets in the original Tai publication, by Gonzales’s method, and HTS-Net, Hotnet2 and CARD algorithms.
(XLSX)
